# Integrated micro/messenger RNA regulatory networks in essential thrombocytosis

**DOI:** 10.1371/journal.pone.0191932

**Published:** 2018-02-08

**Authors:** Lu Zhao, Song Wu, Erya Huang, Dimitri Gnatenko, Wadie F. Bahou, Wei Zhu

**Affiliations:** 1 Department of Applied Mathematics and Statistics, Stony Brook University, Stony Brook, NY, United States of America; 2 Department of Medicine, Stony Brook University, Stony Brook, NY, United States of America; University of São Paulo, BRAZIL

## Abstract

Essential thrombocytosis (ET) is a chronic myeloproliferative disorder with an unregulated surplus of platelets. Complications of ET include stroke, heart attack, and formation of blood clots. Although platelet-enhancing mutations have been identified in ET cohorts, genetic networks causally implicated in thrombotic risk remain unestablished. In this study, we aim to identify novel ET-related miRNA-mRNA regulatory networks through comparisons of transcriptomes between healthy controls and ET patients. Four network discovery algorithms have been employed, including (a) Pearson correlation network, (b) sparse supervised canonical correlation analysis (sSCCA), (c) sparse partial correlation network analysis (SPACE), and, (d) (sparse) Bayesian network analysis–all through a combined data-driven and knowledge-based analysis. The result predicts a close relationship between an 8-miRNA set (miR-9, miR-490-5p, miR-490-3p, miR-182, miR-34a, miR-196b, miR-34b*, miR-181a-2*) and a 9-mRNA set (CAV2, LAPTM4B, TIMP1, PKIG, WASF1, MMP1, ERVH-4, NME4, HSD17B12). The majority of the identified variables have been linked to hematologic functions by a number of studies. Furthermore, it is observed that the selected mRNAs are highly relevant to ET disease, and provide an initial framework for dissecting both platelet-enhancing and functional consequences of dysregulated platelet production.

## 1. Introduction

Platelets are anucleate blood cells generated from bone marrow megakaryocytes, and play an important role in haemostasis and thrombosis. Thrombocytosis is a disorder of platelet overproduction in the blood. It is classified as essential/primary thrombocytosis (ET) or reactive/secondary thrombocytosis (RT) by the causes. Essential thrombocytosis is a chronic myeloproliferative disorder with an unregulated surplus of platelets attributed to a malfunction in the body’s feedback system. Complications of ET include stroke, heart attack, and formation of blood clots. Mutations involving *JAK2*, *CALR*, and *c-MPL* are identified in the majority of ET cohorts, although genetic risk substratification associated with thrombotic (or hemorrhagic) predisposition remains unknown [1].

Recent data have demonstrated that both megakaryocytes and platelets retain an abundant and diverse array of mRNAs and microRNAs (miRNAs) [2]. miRNAs are a class of non-coding 21- to 24-bp species that primarily regulate protein translation by post-transcriptional targeting of 3’—UTRs [3], which subsequently regulates mRNA translation activity or stability [4]. Emerging evidence has implicated miRNAs in the control of megakaryocytopoiesis [5] and in progenitor fate during the megakaryocyte-erythroid transition. Distinct miRNA expression patterns have been described in differentiated hematopoietic cells [6] and in subsets of patients with myeloproliferative neoplasms [7, 8]. The miRNAs have effects on protein synthesis through regulating mRNA destabilization or translational repression [4]; indeed although quiescent platelets display minimal translational activity, maximally-activated platelets retain the capacity for protein synthesis, with implications for modulating arthritis-associated inflammation [9] or the production of platelet progeny *in vivo* [10].

Many computational methods have been developed to study interactions between miRNA and mRNA, which are largely based on two types of methods: one is computation-based method that uses the sequence complementarities of miRNA and its mRNA targets to build *in silicon* interaction databases, including MiRBase [11], TargetScan [12, 13] and so on; the second is experimental data-based method that examines expression profiles of miRNAs and mRNAs for negative correlations. For example, GenMiR++ [14, 15] and HOCTAR [16] predicts the interaction between miRNA and mRNA by integrating the expression profiling and sequence-based recognition software. Several other methods that are based solely on expression profile have also been published. Jayaswal et al. [17] developed a two-stage procedure that first clusters each expression data for miRNA and mRNA and then identify significant miRNA-mRNA relationship using t-test. Li et al. [18] proposed a method to find a set of differentially expressed miRNAs and mRNAs via Partial Least Squares Regression. It is very challenging to build causal relationship using observational data. Le et al. [19] designed an algorithm to uncover the causal regulatory relationship between miRNAs and mRNAs, using expression profiles of miRNAs and mRNAs without taking into consideration the previous target information. It is based on Intervention calculus when the Directed Acrylic Graph (DAG) is absent (IDA) [20]. While all the above methods focus on uncovering interaction between individual miRNA and mRNA, there is a growing body of literature showing that multiple miRNAs are coordinated by forming cohesive groups to collectively regulate one or more mRNAs [21]. The complex regulatory network formed between a group of miRNAs and a group of mRNAs acts as a vital force in catering similar functioning miRNAs and mRNAs together, and may provide better understandings on the underlying miRNA-mRNA regulatory modules (MMRMs) [22].

In this study, we explore the potential miRNA/mRNA regulatory networks associated to essential thrombocytosis based on a 43-member cohort (13 ET patients and 30 controls), through a combination of data-driven and knowledge-based analyses. Three classes of correlation network analyses methods, namely, the Pearson correlation network, the sparse canonical correlation network, and the sparse partial correlation network have been implemented, compared and integrated to obtain a more reliable and robust miRNA-mRNA pathway. This pathway was subsequently examined for its biological functionalities through an Ingenuity Pathway Analysis. Additionally, we have applied a sparse Bayesian Network analysis, the A* Lasso, to compare with the three Frequentist network analysis methods.

## 2. Methods

### 2.1 Patient recruitment, sample processing and data description

Subject recruitment (along with normal healthy controls) was completed by written consent through a study approved by the Stony Brook IRB (Institutional Review Board) Committee on Research Involving Human Subjects (approval period 1999 –present). Enrollment proceeded over a 3-year period and was restricted to adults (>21 years of age) meeting clinical and laboratory criteria for essential thrombocytosis as previously described (38). Patients were randomly enrolled from the larger pool of patients referred for evaluation of thrombocytosis, and the primary ineligibility criteria were failure to provide consent; subject data are from the initial recruitment with no reentry to date. ET is rare in minors and no minors were included in this study. Subject gender distribution (9 females, 4 males) was designed to parallel the relative female preponderance of the disease; healthy controls identified from the ethnically diverse population of Long Island, NY were not matched with thrombocytosis cohorts, but were gender-equivalen (i.e. 15 females, 15 males). Methods for platelet isolation, sample processing, and sample quality control using highly-enriched peripheral blood platelets have been previously described [2, 23–25]. The miRNA data were obtained from sample hybridization to the Agilent G4470C human miRNA gene chip that incorporates 866 human and 89 viral miRNAs (miRBase database Version 12.0) and have been deposited into the public GEO database (GEO accession number GSE39046) [25]. The mRNA data were obtained from a custom 432-member oligonucleotide gene chip specifically designed to characterize human platelet-restricted gene expression data [24], and are publicly available (GEO accession number GSE12295).

In the remaining part of this section, we first introduce the Frequentist network analysis methods used in this study. Subsequently we present the integrated analysis combining the results from these different methods.

### 2.2 Sparse supervised canonical correlation analysis

Introduced by Hotelling in 1936 [26], (the first) canonical correlation between two variable sets looks for the weighted combination of all variables within each variable set such that the correlation of the two combinations is maximized. The weighted combinations are called canonical variables or components. Considering an *n* * *p* matrix *X* and an *n* * *q* matrix *Y*. Without loss of generality, we assume *p* < *q*. Canonical correlation analysis (CCA) [26] seeks coefficient vectors ***u*** and ***v***, such that the correlation between the linear combinations *ω* = ***u***′*X* and *ξ* = ***v***′*Y* is maximized, i.e.

$$\underset{u,v}{\max\;}Corr(\omega ,\xi )=\underset{u,v}{max}\frac{u^{\prime }\Sigma_{XY}v}{\sqrt{u^{\prime }\Sigma_{XX}u}\sqrt{v^{\prime }\Sigma_{YY}v}}$$

where Σ_*XX*_, Σ_*YY*_, and Σ_*XY*_ are the variance for *X*, *Y*, and the covariance for *X* and *Y*, respectively. It is attained by the canonical variate pairs

$$\omega =u^{\prime }X=e^{\prime }{\Sigma_{XX}}^{-\frac{1}{2}}X;\;\xi =v^{\prime }Y=f^{\prime }{\Sigma_{YY}}^{-\frac{1}{2}}Y$$

with *e* and *f* from the singular value decomposition (SVD) of the matrix *K* given by $K={\Sigma_{XX}}^{-\frac{1}{2}}\Sigma_{XY}{\Sigma_{YY}}^{-\frac{1}{2}}=eDf^{\prime }$ [27].

In canonical correlation analysis, all variables are included in the linear combinations, yet for genetic data obtained via microarray studies or other high throughput methods, the number of variables usually surpasses tens of thousands, far exceeding the number of study subjects. Thus the fitted linear combinations may not be easily interpreted and the application of standard algorithms may fail. These problems can be solved by introducing sparse loadings in the canonical components, i.e. the sparse canonical correlation analysis (SCCA) proposed in 2007 [27]. The idea of SCCA is consistent with the belief that only a modest set of genes are truly associated with a given trait of interest.

Based on the foundation of SCCA, Witten and Tibshirani [28] further presented “sparse supervised canonical correlation analysis (sSCCA)”, targeting on finding the sparse linear combinations of the two variable sets that are correlated with each other and also associated with the trait of interest. Still considering an *n* * *p* matrix *X* and an *n* * *q* matrix *Y*, and assuming that the columns of *X* and *Y* have been standardized with mean 0 and standard deviation 1. Suppose in addition we have a categorical outcome vector $z\in R^{n}$. The estimates of canonical vectors are defined as

$$\max_{u,v}u^{T}X^{T}Yv,\;\mathrm{subject}\;\mathrm{to}$$

$$\|u{\|}^{2}\leq 1,\;\|v{\|}^{2}\leq 1,\;P_{1}(u)=\|u{\|}_{1}\leq c_{u},\;P_{2}(v)=\|v{\|}_{1}\leq c_{v},$$ (1)

$$u_{j}=0\forall j\notin Q_{u},\;v_{j}=0\forall j\notin Q_{v},$$

where *P*_1_ and *P*_2_ are convex penalty functions; *c*_*u*_ and *c*_*v*_ are assumed to be ${1\leq c}_{u}\leq \sqrt{p}$ and ${1\leq c}_{v}\leq \sqrt{q}$; *Q*_*u*_ and *Q*_*v*_ are the sets of variables with highest univariate association with the outcome *z* in *X* and *Y*, respectively; the threshold for variables to be included in *Q*_*u*_ and *Q*_*v*_ can either be fixed or defined as tuning parameters. The vectors *u* and *v* are obtained using an iterative algorithm with soft-thresholding. We have performed this sSCCA method on our genetic data set to investigate whether the expression of miRNA would have a significant effect on that of genes and vice versa.

### 2.3 Sparse partial correlation analysis

Given *p* continuous random variables {*X*_*i*_, *i* = 1,2,…, *p*}` from *n* samples, we can denote the set of measurements/data as

$$X={\left(X_{1},X_{2},\ldots \;\ldots ,X_{P}\right)}^{T}\;\in R^{n\times p}$$

Here the rows of the matrix represent the samples and the columns the variables. Within each column (variable), the data are centered to the column mean. For any two random variables *X*_*i*_ and *X*_*j*_, we denote the set of all other variables as *X*_−(*i*,*j*)_, that is,

$$X_{-(i,j)}=X\setminus \{X_{i},X_{j}\}=\{X_{k},1\leq k\neq i,j\leq p\}$$

where *X*_*i*_ and $X_{j}\in R^{n}$ are the *i*th and *j*th columns of *X*, and $X_{-(i,j)}\in R^{n\times (p-2)}$ is the matrix obtained from *X* by deleting its *i*th and *j*th columns. Without loss of generality, we assume that *i* < *j*.

The Sparse partial Correlation Analysis (SPACE) is a modern method for estimating the partial correlation coefficient also relates to the least square regression problem [29]. This method starts with constructing *p* linear regression models

$$X_{i}=X_{-\left(i\right)}\beta^{\left(i\right)}+\varepsilon_{i}=\sum_{k\neq i}\beta_{k}^{\left(i\right)}X_{k}+\varepsilon_{i},i=1,2,\ldots ,p$$ (2)

where *ε*_*i*_ are i.i.d. disturbance terms, the least square estimate of the regression coefficient vector is calculated as

$$\begin{array}{c} {\hat{\beta }}^{(i)}=\left({\hat{\beta }}_{1}^{\left(i\right)},{\hat{\beta }}_{2}^{\left(i\right)},\ldots ,{\hat{\beta }}_{i-1}^{\left(i\right)},{\hat{\beta }}_{i+1}^{\left(i\right)},\ldots ,{\hat{\beta }}_{p}^{(i)}\right)=\arg{\underset{\beta \in R^{p-1}}{\min}\left\|X_{i}-X_{-(i)}\beta \right\|}^{2}\\ ={\left(X_{-(i)}^{T}X_{-(i)}\right)}^{-1}X_{-(i)}^{T}X_{i},\;for\;i=1,2,\ldots ,p \end{array}$$

The sample partial correlation coefficient is then estimated as ${\hat{\rho }}_{ij}=sign\left({\hat{\beta }}_{j}^{\left(i\right)}\right)\sqrt{{\hat{\beta }}_{j}^{\left(i\right)}{\hat{\beta }}_{i}^{\left(j\right)}}$.

### 2.4 Sparse Bayesian network analysis

The fundamental structure among a series of random variables is depicted by their joint probability distribution. Probabilistic graphical models are used to describe the conditional independence or dependence structure implied by the joint distribution with a graph-induced decomposition of the joint density function. A Bayesian Network (BN), a branch of probabilistic graphical model, is a probabilistic graphical model defined over a DAG *G* with a set of *p* = |*V*| nodes *V* = {*v*_1_, ⋯, *v*_2_}. In such a graph or network, a node is a random variable, and an edge between two nodes indicates certain stochastic association. The probability model associated with *G* in a Bayesian network factorizes as $p(X_{1},\cdots ,X_{p})=\prod_{j=1}^{p}p(X_{j}|Pa(X_{j}))$, where *p*(*X*_*j*_|*Pa*(*X*_*j*_)) is the conditional probability distribution for *X*_*j*_ given its parents *Pa*(*X*_*j*_) with directed edges from each node in *Pa*(*X*_*j*_) to *X*_*j*_ in *G*. For Gaussian random variables, conditional independence of X and Y given Z is equivalent to a zero partial correlation: *ρ*_*XY*⋅ *Z*_ = 0. This provides certain insight into the relationship between the Bayesian network and the partial correlation network in that, the partial correlation, by controlling all other variables except the two targeting variables, should in general be more conservative than the Bayesian network.

A recently published paper [30] presented an algorithm entitled A* lasso, for learning a Sparse Bayesian Network structure for continuous variables in a high-dimensional space. Compared to the common two-stage inference methods, A* lasso is a single stage method that recovers the optimal sparse Bayesian network structure by solving a single optimization problem with A* search algorithm that uses lasso in its scoring system. The A*lasso method assumes continuous random variables and uses a linear regression model for the conditional probability distribution of each node *X*_*j*_ = *Pa*(*X*_*j*_) * *β*_*j*_ + *ϵ*, where $\beta_{j}=\{\beta_{jk}^{\prime }sfor\;X_{k}\in Pa(X_{j})\}$ is the vector of unknown parameters to be estimated from data and *ϵ* is the noise distributed as *N*(0, 1). The BN’s structure and parameters are obtained by minimizing the negative log likelihood of data with sparsity enforcing *L*_1_ penalty as follows:

$$\underset{\beta_{1},\cdots ,\beta_{p}}{\min}\sum_{j=1}^{p}{\left\|x_{j}-x_{-j}^{\prime }\beta_{j}\right\|}_{2}^{2}+\lambda \sum_{j=1}^{p}{\left\|\beta_{j}\right\|}_{1}\;s.t.G\in DAG,$$ (3)

where *X*_−*j*_ represents all columns of ***X*** excluding *x*_*j*_, assuming all other variables are candidate parents of node *v*_*j*_.

This lasso optimization problem can be solved efficiently with the shooting algorithm [31] if the acyclicity constraint is ignored, which is the most challenge part of the BN inference procedure. A heuristic scheme of A* lasso is proposed to prune search space when learning the Bayesian network structure by exploring a scoring algorithm based on lasso score generated by the shooting algorithm *f*(*Q*_*s*_) = *g*(*Q*_*s*_) + *h*(*Q*_*s*_) [31]. Here *Q*_*s*_ is the set of variables for which the ordering has been determined. And *g*(*Q*_*s*_) is the accumulated cost for reaching the *Q*_*s*_ state:

$$g(Q_{s})=\sum_{v_{j}\in Q_{s}}LassoScore(v_{j}|\prod_{<v_{j}}^{Q_{s}}h(Q_{s}))$$ (4)

*Here h*(*Q*_*s*_) is the estimated cost of reaching the goal stat from the current state

$$g(Q_{s})=\sum_{v_{j}\in {V\backslash Q}_{s}}LassoScore(v_{j}|V\backslash v_{j})$$ (5)

Furthermore, the Lasso Score is defined as

$$LassoScore(v_{j}|V\backslash v_{j})=\min_{\beta_{j}}{\left\|x_{j}-x_{-j}^{\prime }\beta_{j}\right\|}_{2}^{2}+\lambda \sum_{j=1}^{p}{\left\|\beta_{j}\right\|}_{1}$$ (6)

On top of the heuristic scheme, A* lasso further reduces the search space by limiting the size of intermediate search path via a size-limited priority queue that orders the promising intermediate search paths via the above scoring scheme. The combined strategy gives the A* lasso great advantage in efficiency over the common DP algorithms, which makes it scalable for high-dimension data, such as the miRNA and mRNA interaction problem in our study.

### 2.5 A novel joint network analysis pipeline

We proposed a novel pipeline for extracting miRNA and mRNA interaction network by combining the sSCCA and the SPACE methods. Our pipeline is designed for small/moderate sample size with large number of miRNAs and mRNAs. In order to extract meaningful insights from small/moderate datasets, the pipeline selects most relevant miRNAs and mRNAs that has the largest canonical correlation via sSCCA and then identifies links between these selected miRNAs and mRNAs through the SPACE method, where the latter would compute the pair-wise partial correlation coefficient conditioned on other features.

**Fig 1:**
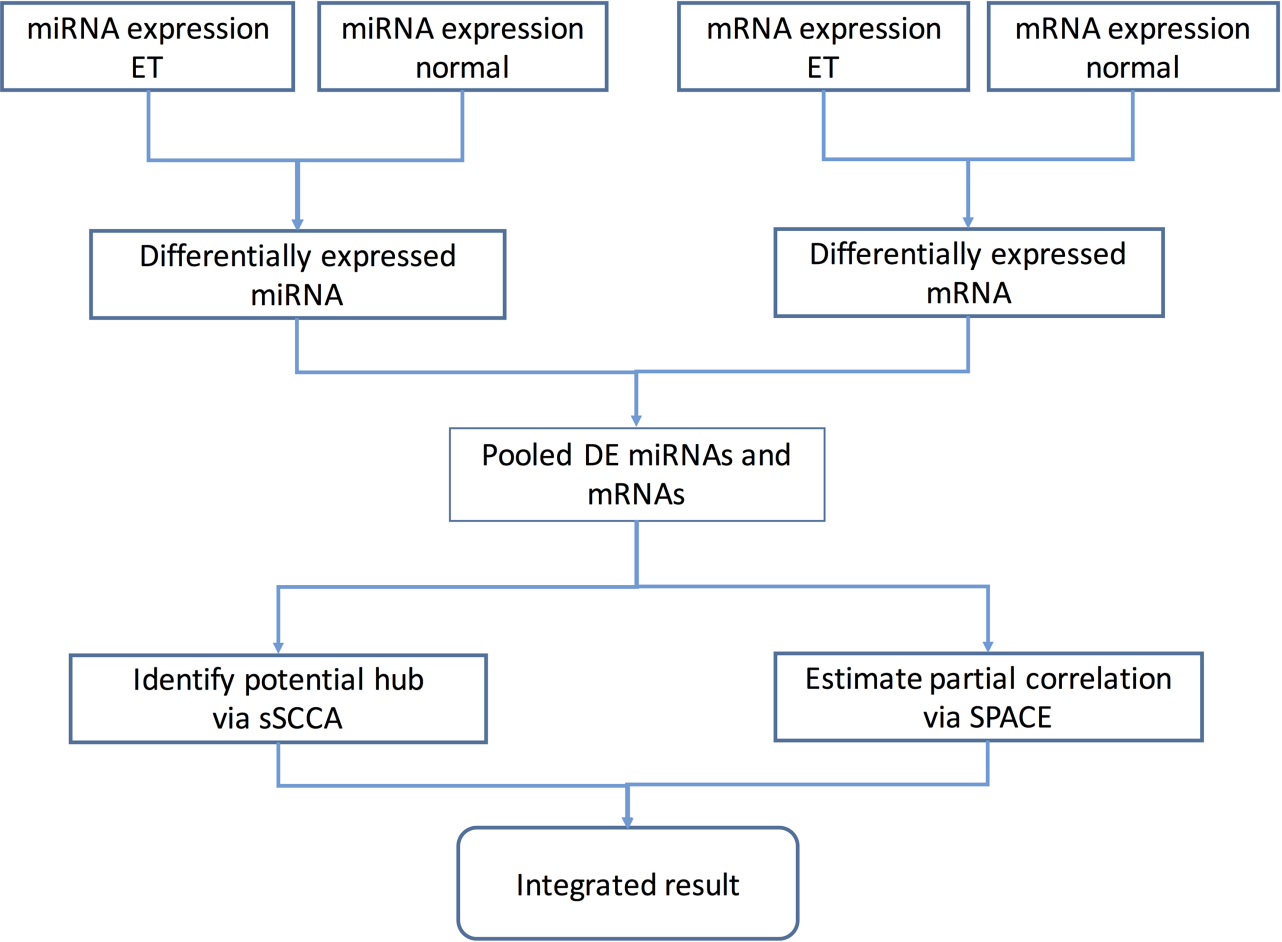
Pipeline of extracting the data-based miRNA and mRNA interaction networks through the joint sparse supervised canonical correlation network analysis (sSCCA) and sparse partial correlation network analysis (SPACE).

There are four steps in the pipeline (Fig 1). First, the differentially expressed (DE) miRNAs and mRNAs are selected via either limma [32] or SAM [33], which are commonly used methods for DE detection. Second, a subset of miRNAs and mRNAs are selected by performing the sSCCA method on the pooled DE miRNAs and mRNAs. In the third step, the pair-wise partial correlations are calculated by performing SPACE on the pooled DE miRNAs and mRNAs. Lastly, only the links that connects the selected miRNAs and mRNAs by sSCCA are kept and added to the sSCCA result.

## 3. Results

### 3.1 Data structure and processing

Our study integrated platelet mRNA/miRNA expression data from two distinct data sets: (1) mRNA expression data were obtained using a 432-member platelet-specific oligonucleotide custom array as previously described [24], and (2) miRNA expression data were obtained from sample hybridization to the Agilent G4470C human miRNA gene chip that incorporates 866 human and 89 viral miRNAs (miRBase database Version 12.0) [25]. Both mRNA and miRNA expression levels have been collected on 13 patients with essential thrombocytosis (ET) disease and 30 control subjects (S1 Table). Subject recruitment (along with normal healthy controls) was completed by written consent through a study approved by the Stony Brook IRB (Institutional Review Board) Committee on Research Involving Human Subjects (CORIHS), and was restricted to adults (>21 years of age) meeting clinical and laboratory criteria for essential thrombocytosis as previously described (38). Subject gender distribution (9 females, 4 males) paralleled the relative female preponderance of the disease; healthy controls were matched by gender (i.e. 15 females, 15 males).

Among 43 samples, there are 7 (3 ET, 4 NO) samples that have two technical replicates. The values of these samples are reset by the mean value of the sample replicates. The original miRNA data set was filtered in two steps: The first step is to filter out miRNAs with less than 30% non-absent cells in both groups. Next, miRNAs with more than 40% missing values in the sample sets were also dropped out. For the mRNA data, the proportion of missing expression data in the sample set for each mRNA was calculated and those with 50% or more absent data have been excluded. In addition, potential outliers were checked and filtered with a criterion of 3 standard deviations from the mean expression value. In both data sets, quantile normalization was applied to correct the between-array variation [34]. There are 93 out of 432 genes that have missing values in at least one sample. In general, it leads to selection biases if the missing values are simply discarded or the corresponding genes are removed; We decided to impute the missing values using the k-nearest neighbors algorithm [35] implemented in the *impute* R package, which takes into the consideration the correlation structure of the data.

After data filtering and processing, there are totally 327 platelet-specific mRNAs and 396 miRNAs left. To identify highly DE miRNAs and mRNAs, Linear models for microarray data (limma) [36] was applied to the expression data and design matrix. After fitting the linear model, the standard errors are moderated using a simple empirical Bayes model using eBayes function in limma package. Then top DE miRNAs and mRNAs are selected based on the adjusted p-value for the coefficient/contrast of interests. A total of 61 miRNAs and 19 mRNAs were selected at the significant level 0.01 adjusted by the Benjamini-Hochberg (BH) method.

### 3.2 Individual and combined network analysis results

With the 61 selected miRNAs as one variable set, the 19 mRNAs as the other, and the vector of subject disease status as a binary outcome vector, we applied four network analysis methods (Pearson correlation, sSCCA, SPACE and the Bayesian A* lasso) to the differentially expressed (DE) data sets (miRNA and mRNA).

On the Pearson correlation analysis, the pair-wise Pearson correlation coefficient is calculated using the “psych” R package, and 3164 non-zero coefficients are identified at the significant level 0.01 adjusted by the BH method. It covers all links from the results of SPACE and A* lasso, which indicates that the Pearson correlation may generate much more false positives than the other methods. Therefore, we have decided to focus on the results of the other three methods.

**Fig 2:**
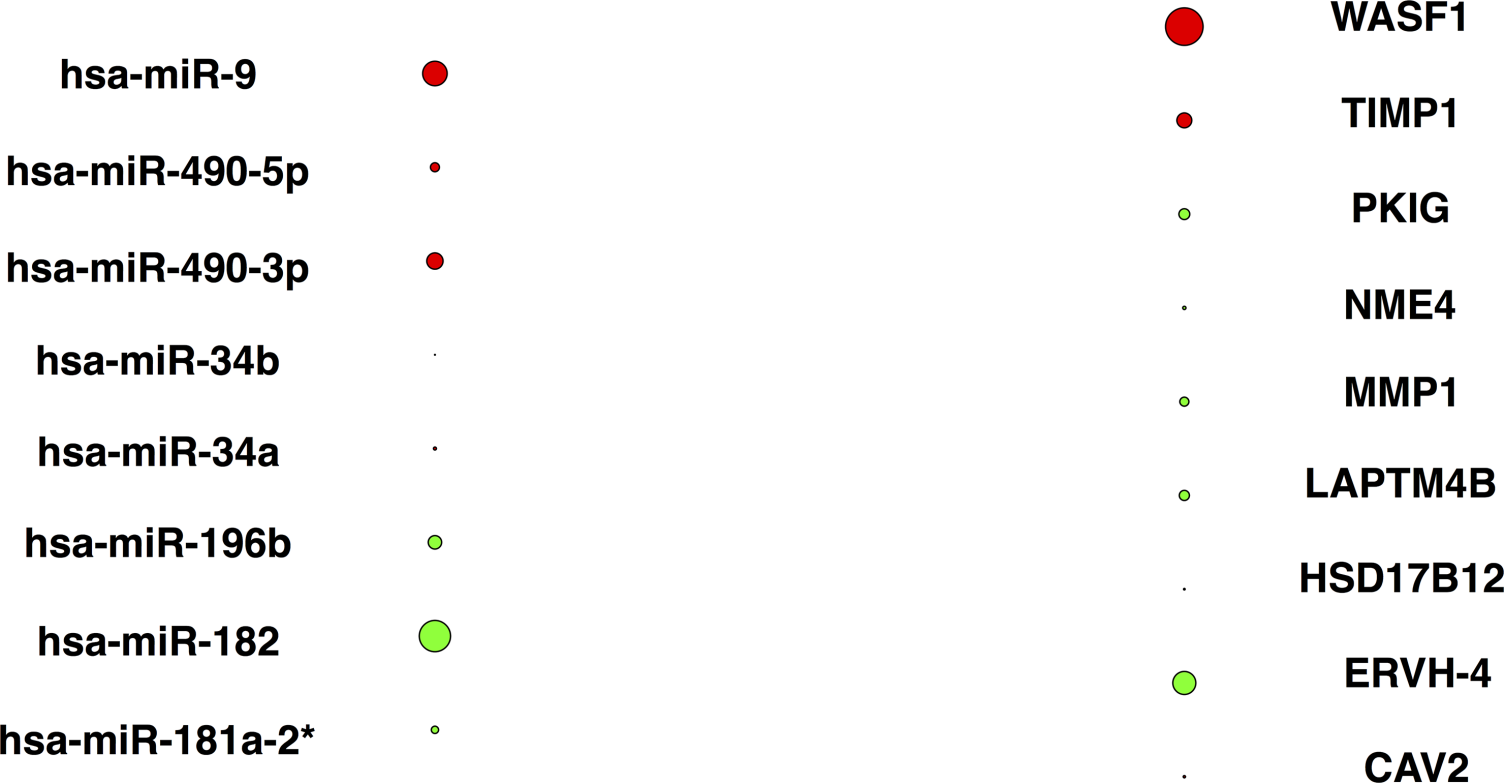
Bipartite plot of the sSCCA result. Red or green node represents positive or negative weight in vector *u* and *v*. The node size represents the absolute value of weight.

On the sSCCA method, the miRNA and mRNA subsets were selected with the penalty of 0.3 (default value in R package SPACE) on vector *u* and 0.5 on vector *v*. As discussed previously, vector *u* restricts the number of selected miRNA, while vector *v* does the same to the mRNA. In the result, 8 miRNAs stand out with 9 corresponding mRNAs. Fig 2 visualizes the weights in the loadings of the first canonical correlation coefficient of selected miRNAs and mRNAs. The actual values are tabulated in supplementary S2 Table.

SPACE is a penalized method, which has one tuning parameter that controls the *L*_1_ penalty on Lasso regression. The value is set as 0.5765849 as calculated by the following equation.

$$L_{1}=\frac{\Phi (1-\frac{\alpha }{2*p^{2}})}{\sqrt{n}}$$ (7)

Here *n* is the sample size (43), *p* is the number of features (80), and *α* is a constant (1).

**Fig 3:**
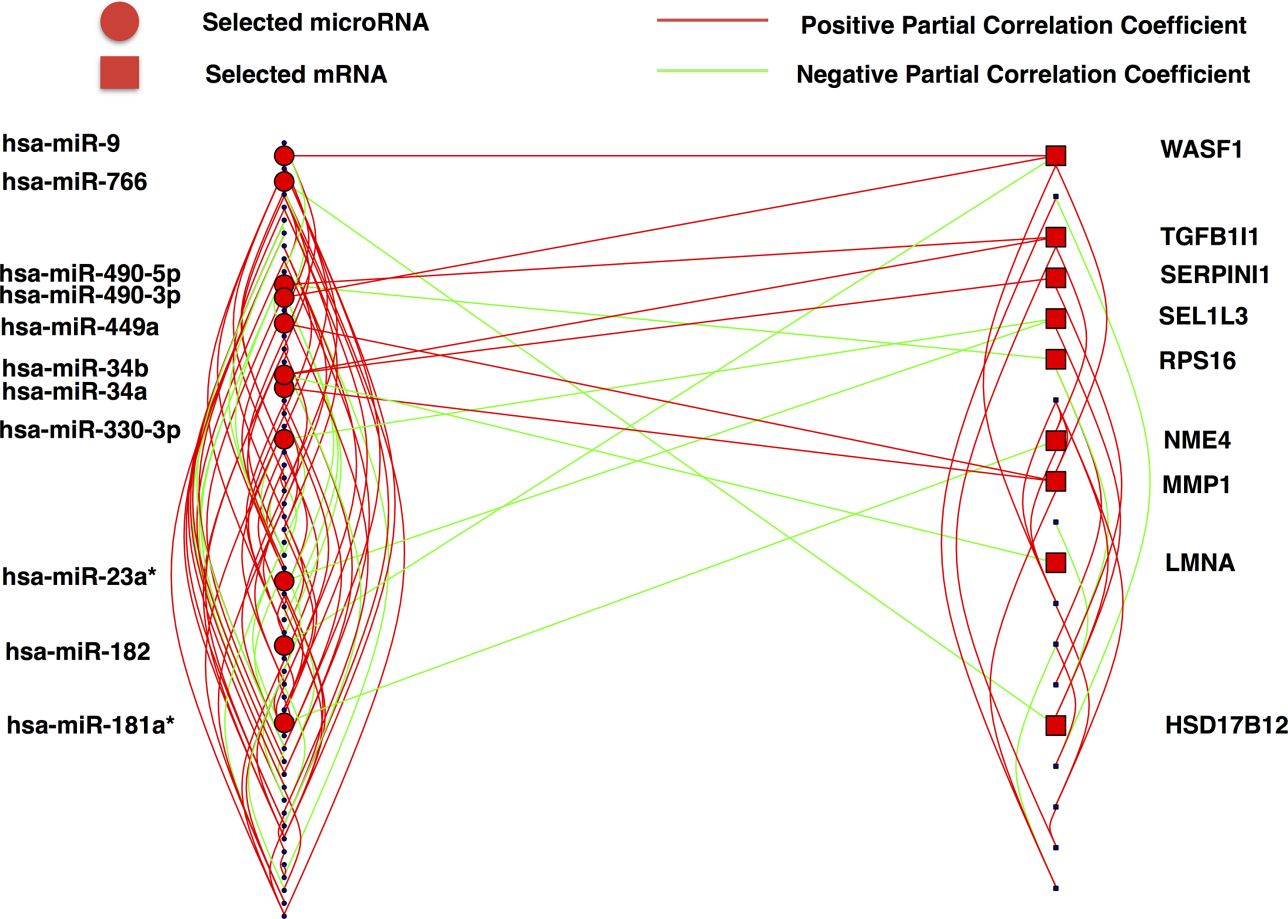
Bipartite plot of the SPACE result. Red circles represent miRNAs that have direct connection with the mRNAs, while the red squares denote the mRNAs that have direct link with the miRNAs. In addition, red and green lines represent positive or negative partial correlations between the pairs.

Fig 3 illustrates the SPACE interaction network emphasizing the interactions between miRNAs and mRNAs. Those miRNAs and mRNAs that have direct links with each other are labeled. The network is connected and there is no isolated node. Within-group links accounts for most of the edges of the network, suggesting that interaction within group is more common than that between groups. There are only 14 (14/165) direct links between miRNAs and mRNAs.

On the result from the A* lasso algorithm, there are two critical parameters. One is the *L*_1_ penalty on Lasso regression. We chose 0.2 (recommended value) as the *L*_1_ value. The other parameter is the queue size that limits the search depth. In order to obtain a near optimal structure, 3,000 is chosen for this option. Since all mRNAs have direct links with miRNAs, the names are not listed in the figure (S1 Fig), A* lasso shows the same pattern as the SPACE result, namely, within group interaction is more common than between group interaction.

**Fig 4:**
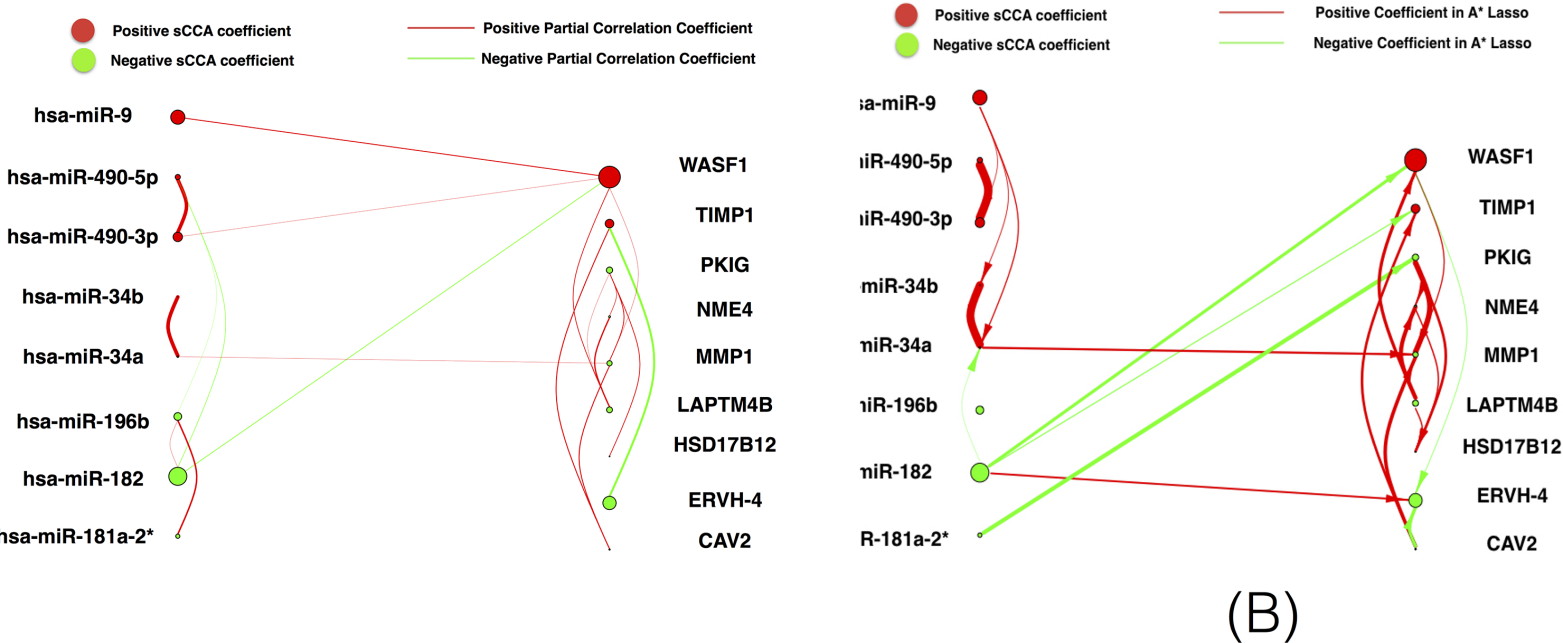
Integrated network analysis results. (A) is the integrated result between sSCCA and SPACE. (B) is the integrated result between sSCCA and A* Lasso method. The arrow is added back on figure (B). The red represents positive values (either weight or correlation coefficient) and green means negative values.

A* lasso identified 306 links that covers 192 out of 250 links from SPACE result, which is consistent with our expectation that SPACE should be more conservative than A* lasso considering the methodological differences. Since it is very hard to interpret a network with too many links and nodes, we integrate the SPACE and A* lasso result with result from sSCCA by only keeping the selected miRNAs, mRNAs and the corresponding links from SPACE and A* lasso method (Fig 4) respectively. Fig 4 compares the integrated results using SPACE and A* Lasso method with sSCCA. The interaction within the selected mRNAs are strikingly consistent both on links and the value signs except A* Lasso has more links. Two miRNA and mRNA interactions are overlapped. One is the link between has-miR-182 and WASF1. The other is the link between has-mir-34a and MMP1 gene (miRNA).

To render the results more comprehensive, the expression value of those selected miRNAs and mRNAs are tabulated in S3 and S4 Tables. All selected miRNAs and mRNAs are differentially expressed with very small adjusted p-values (all less than 0.0001).

## 4. Discussion

In this paper, we proposed a new integrative approach that extracts miRNA and mRNA interaction network by combining the sSCCA and the SPACE methods. Compared to the widely used methods (see S5 Table for detail comparison), such as HOCTAR and GenMiR++, our pipeline is designed for small/moderate sample size with large number of miRNAs and mRNAs and only focuses on the most relevant and sparse networks.

Our joint network analyses using miRNA and mRNA expression data have predicted a close relationship between 8 miRNAs (including miR-9, miR-490-5p, miR-490-3p, miR-182, miR-34a, miR-196b, miR-34b*, miR-181a-2*) and a 9-mRNA set (including CAV2, LAPTM4B, TIMP1, PKIG, WASF1, MMP1, ERVH-4, NME4, HSD17B12), collectively implicating distinct miRNA/mRNA subsets in an integrated network regulating the essential thrombocythemia (*vide infra*). The ET phenotype encompasses two distinct biological pathways, specifically (1) a regulatory network that controls excess platelet production either by effecting megakaryocyte proliferation or proplatelet formation, and (2) a presumably disconnected network that affects platelet functional activity leading to thrombotic or hemorrhagic risk known to accompany ET [37]. Despite these dichotomous functions, molecular defects causally implicated in platelet-associated bleeding or thrombosis remain largely unknown, sharply contrasting with genetic regulation of hematopoietic proliferation/differentiation signals known to accompany terminal megakaryocytopoiesis and platelet production. Application of our miRNA/mRNA network to platelet functional responses provides a logical framework for subsequent delineation of clinical thrombohemorrhagic outcomes in defined ET cohorts.

Notably, the network(s) identified by SPACE and A* lasso methods have significant overlap, serving to validate our conclusions by applying distinct approaches to yield comparable results. Two overlapped links (*miR-182*-*WASF1* and *miR-34a*-*MMP1*) are worthy targets for biological validation since all four mRNAs/miRNAs have been previously implicated in the ET phenotype. Indeed, *miR-34a* and *miR-182* identified by sparse SCCA have been previously described as demonstrating aberrant expression in polycythemia vera (PV) granulocytes [8]; furthermore, both *miR-34a* and *miR-182* are among the most significant differentially-expressed miRNA members among a cohort of thrombocytosis subjects [25]. The *miR 34* family members (*miR 34a* and miR *34b/c*) contain p53 binding sites, and *miR 34a* is widely studied as a tumor suppressor gene and as a potential therapeutic target in human cancer [38]. No prior evidence has demonstrated that *miR34a* regulates *MMP1* (matrix metalloproteinase 1) as demonstrated by our data [38]. Indeed, both *MMP1* and its inhibitor *TIMP1* (tissue inhibitor of metalloproteinases 1) are members of a well-characterized class of proteinases involved in tumor invasiveness and cancer metastases [39]. Furthermore, *TIMP1* has been predicted as a putative *miR-34a* target using the target prediction tools TargetScan [40], designed to identify regulatory targets using conserved complementary [12]. Members of the matrix metalloproteinase family have been implicated in the migration and invasion of leukemia cell (*MMP-2*) [41], and previously shown to mediate megakaryocyte transendothelial migration and proplatelet formation (*MMP-9*) [42]. *MMP1* has also been studied in the context of inflammation in several studies [43–46], thereby providing an additional link to the known function(s) of platelets in adaptive immunity [37].

In addition to *MMP1/TIMP1*, various other transcripts within the 9-member mRNA list have critical roles in platelet biology and function. Indeed, both *CAV2* (caveolin 2) and *WASF1* (WAS protein family, member 1) have fundamentally important functions in maintaining cytoskeletal function and viability of membrane/lipid rafts, key regulators of the platelet activation response. Moreover, the WAS protein family has been shown to be related to nucleosome and chromatin assembly, performing an important role in gene transcription that may regulate megakaryocytopoiesis and/or proplatelet formation [47]. A recent study in class prediction models of ET included a member from this family (*WASF3*) as one of the biomarkers segregating ET from reactive thrombocytosis and healthy controls [24], thereby extending the role of the WAS family of proteins in key regulatory functions of megakaryocytopoiesis and/or platelet activation. Finally, *HSD17B12* (hydroxysteroid (17-β) dehydrogenase 12) which catalyzes the penultimate step in testosterone synthesis, has been previously identified as a functionally-active dehydrogenase in ET platelets, serving as a putative link to gender-regulated differences in platelet function [23].

We also used the Ingenuity Pathway Analysis (IPA) software to further characterize the confirmed associations between 8-miRNAs and 9-mRNAs. IPA predicts that *miR-9* and *miR-196b* have interaction with *NME4* (NME/NM23 nucleoside diphosphate kinase 4) which links to several fundamentally important pathways regulating nucleotide synthesis expected to be active during enhanced megakaryoctyopoiesis (i.e. salvage pathways of pyrimidine ribonucleotides; pyrimidine ribonucleotides *de novo* biosynthesis; pyrimidine ribonucleotides interconversion; pyrimidine deoxyribonucleotides *de novo* biosynthesis 1). It also links miR-34a/miR-34b* with *WASF1* and relates these two links to multiple pathways critical for platelet function (including actin cytoskeleton signaling; actin nucleation by ARP-WASP complex; epithelial adherens junction signaling; Rac signaling; regulation of actin-based motility by Rho; RhoA Signaling; RhoGDI Signaling; and signaling by Rho family GTPases). These pathways are relevant not only to megakaryocyte development and proplatelet formation, but also have fundamental relevance to platelet activation and signaling linked to cardio/cerebrovascular thrombotic diseases.

## Supporting information

S1 Fig Network generated by A* lasso. (TIFF)

S1 Table Data structure. (DOCX)

S2 Table Loadings of miRNA and mRNAs in the first canonical component of sSCCA result. (DOCX)

S3 Table Quantile normalized expression of selected miRNAs. (DOCX)

S4 Table Quantile normalized expression of selected mRNAs. (DOCX)

S5 Table Comparison with HOCTAR and GenMiR++. (DOCX)
