# Supplementary figures and images for: Integrated micro/messenger RNA regulatory networks in essential thrombocytosis
Source: PLoS One. 2018 Feb 8;13(2):e0191932. doi: 10.1371/journal.pone.0191932 (PMC5805260; doi:10.1371/journal.pone.0191932)

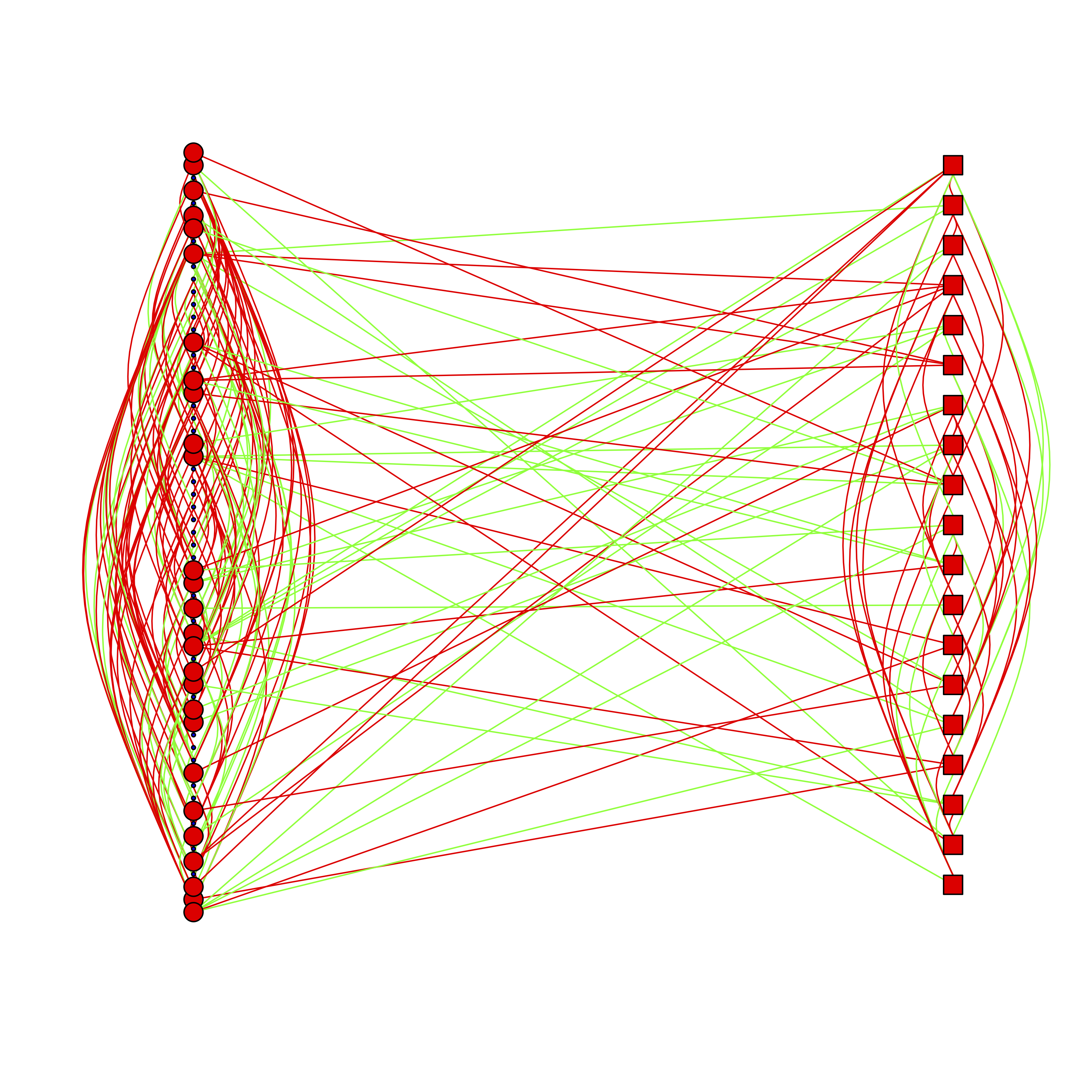

Supplement: S1 Fig — (TIFF) [file pone.0191932.s001.tiff]
