# Supplementary material for: Integrated micro/messenger RNA regulatory networks in essential thrombocytosis
Source: PLoS One. 2018 Feb 8;13(2):e0191932. doi: 10.1371/journal.pone.0191932 (PMC5805260; doi:10.1371/journal.pone.0191932)
Supplement: S1 Table — (DOCX) [file pone.0191932.s002.docx]

S1 Table Data structure

|  | **ET** | **Control** | **Total** |
| --- | --- | --- | --- |
| **# of Paired Subjects** | 13 | 30 | 43 |
|  | **miRNA** | **mRNA** | **Subject** |
| **Total # of Items** | 939 | 354 | 43 |
