# Supplementary material for: Integrated micro/messenger RNA regulatory networks in essential thrombocytosis
Source: PLoS One. 2018 Feb 8;13(2):e0191932. doi: 10.1371/journal.pone.0191932 (PMC5805260; doi:10.1371/journal.pone.0191932)
Supplement: S2 Table — (DOCX) [file pone.0191932.s003.docx]

S2 Table Loadings of miRNA and mRNAs in the first canonical component of sSCCA result

| **miRNA** | **weight** | **mRNA** | **weight** |
| --- | --- | --- | --- |
| Has-miR-9 | 0.609 | WASF1 | 0.797 |
| Has-miR-182 | -0.537 | TIMP1 | 0.442 |
| Has-miR-490-3p | 0.439 | CAV2 | 0.244 |
| Has-miR-490-5p | 0.289 | HSD17B12 | 0.232 |
| Has-miR-196b | -0.171 | NME4 | 0.157 |
| Has-miR-34a | 0.165 | ERVH-4 | -0.154 |
| Has-miR-34b* | 0.089 | MMP1 | 0.066 |
| Has-miR-181a-2* | -0.045 | LAPTM4B | 0.049 |
|  |  | PKIG | 0.038 |
