# Supplementary material for: Integrated micro/messenger RNA regulatory networks in essential thrombocytosis
Source: PLoS One. 2018 Feb 8;13(2):e0191932. doi: 10.1371/journal.pone.0191932 (PMC5805260; doi:10.1371/journal.pone.0191932)
Supplement: S3 Table — (DOCX) [file pone.0191932.s004.docx]

S3 Table Quantile normalized expression of selected miRNAs

| **miRNAs** | **Normal** | | **ET** | | **Log fold change** | **Adjusted p-value** |
| --- | --- | --- | --- | --- | --- | --- |
|  | Mean | std | mean | std |  |  |
| Has-miR-9 | 2.6443 | 0.7643 | 4.8766 | 1.6094 | 2.232166487 | <0.0001 |
| hsa-miR-490-5p | 1.2040 | 1.0495 | 4.7451 | 2.3452 | 3.541056294 | <0.0001 |
| hsa-miR-182 | 3.8207 | 0.7794 | 2.0378 | 1.3189 | -1.782930513 | <0.0001 |
| hsa-miR-34a | 6.0485 | 0.7324 | 8.2727 | 0.9558 | 2.224145999 | <0.0001 |
| hsa-miR-490-3p | 1.1563 | 1.1951 | 5.3272 | 2.3615 | 4.170990216 | <0.0001 |
| hsa-miR-196b | 6.1403 | 0.3886 | 5.2337 | 0.5812 | -0.906563218 | <0.0001 |
| hsa-miR-34b* | 2.1224 | 0.9340 | 4.5740 | 0.9960 | 2.451596604 | <0.0001 |
| hsa-miR-181a-2* | 5.5544 | 0.4086 | 4.4720 | 0.7544 | -1.082323577 | <0.0001 |
