# Supplementary material for: Integrated micro/messenger RNA regulatory networks in essential thrombocytosis
Source: PLoS One. 2018 Feb 8;13(2):e0191932. doi: 10.1371/journal.pone.0191932 (PMC5805260; doi:10.1371/journal.pone.0191932)
Supplement: S4 Table — (DOCX) [file pone.0191932.s005.docx]

S4 Table Quantile normalized expression of selected mRNAs

| **Genes (miRNA)** | **Normal** | | **ET** | | **Log fold change** | **Adjusted p-value** |
| --- | --- | --- | --- | --- | --- | --- |
|  | mean | std | mean | std |  |  |
| CAV2 | 1.2077 | 0.7195 | 2.1356 | 0.7285 | 0.928780454 | <0.0001 |
| LAPTM4B | 0.7808 | 0.3000 | 1.7317 | 1.2278 | 0.989508148 | <0.0001 |
| TIMP1 | 0.8762 | 0.7070 | 2.6453 | 1.5047 | 1.685525634 | <0.0001 |
| PKIG | 0.7111 | 0.2330 | 1.4218 | 0.3989 | 1.031451534 | <0.0001 |
| WASF1 | 0.7378 | 0.2937 | 2.8658 | 1.7532 | 1.694837353 | <0.0001 |
| MMP1 | 0.9536 | 0.8535 | 2.3396 | 1.4260 | 1.28293844 | <0.0001 |
| ERVH-4 | 1.3802 | 0.7963 | 0.6395 | 0.4932 | -1.3561759 | <0.0001 |
| NME4 | 0.7495 | 0.3800 | 1.8671 | 1.3119 | 1.231506643 | <0.0001 |
| HSD17B12 | 0.8473 | 0.6015 | 2.2129 | 0.9899 | 1.535522453 | <0.0001 |
