# Supplementary material for: Integrated micro/messenger RNA regulatory networks in essential thrombocytosis
Source: PLoS One. 2018 Feb 8;13(2):e0191932. doi: 10.1371/journal.pone.0191932 (PMC5805260; doi:10.1371/journal.pone.0191932)
Supplement: S5 Table — (DOCX) [file pone.0191932.s006.docx]

S5 Table Comparison with HOCTAR and GenMiR++

|  | HOCTAR | GenMiR++ | Our suite of method |
| --- | --- | --- | --- |
| Strength | - Applicable when miRNA expression data is not available - Supported by large set of expression data as it uses host gene as proxy of underlying miRNA | - Able to capture one-to-many relationship between mRNA and miRNAs - Leverages the sequence information on top of miRNA-mRNA expression data | - Able to capture many-to-many relationship between mRNAs and miRNAS - Applicable to handle small/moderate data set - Focus on the most relevant and minimal miRNA-mRNA networks |
| Drawbacks | - Cannot capture the many-to-many relationship among multiple miRNAs and mRNAs - Only consider negative correlation between miRNAs and mRNAs | - Not suitable for small/moderate dataset - Convergence rate varies on the likelihood form and prior | - Hard to tune the whole procedure as there are selection thresholds at each step |
